# Speed and Cardiac Recovery Variables Predict the Probability of Elimination in Equine Endurance Events

**DOI:** 10.1371/journal.pone.0137013

**Published:** 2015-08-31

**Authors:** Mohamed Younes, Céline Robert, François Cottin, Eric Barrey

**Affiliations:** 1 UBIAE, Université d’Evry Val d’Essonne, Evry, France; 2 INRA, GABI, UMR1313, Jouy-en-Josas, France; 3 Université Paris-Est, Ecole Nationale Vétérinaire d'Alfort, Maison Alfort, France; 4 EA4532 CIAMS, Université Paris Sud, Orsay, France; 5 Département STAPS, Université d’Evry Val d’Essonne, Evry, France; University of Minnesota, UNITED STATES

## Abstract

Nearly 50% of the horses participating in endurance events are eliminated at a veterinary examination (a vet gate). Detecting unfit horses before a health problem occurs and treatment is required is a challenge for veterinarians but is essential for improving equine welfare. We hypothesized that it would be possible to detect unfit horses earlier in the event by measuring heart rate recovery variables. Hence, the objective of the present study was to compute logistic regressions of heart rate, cardiac recovery time and average speed data recorded at the previous vet gate (n-1) and thus predict the probability of elimination during successive phases (n and following) in endurance events. Speed and heart rate data were extracted from an electronic database of endurance events (80–160 km in length) organized in four countries. Overall, 39% of the horses that started an event were eliminated—mostly due to lameness (64%) or metabolic disorders (15%). For each vet gate, logistic regressions of explanatory variables (average speed, cardiac recovery time and heart rate measured at the previous vet gate) and categorical variables (age and/or event distance) were computed to estimate the probability of elimination. The predictive logistic regressions for vet gates 2 to 5 correctly classified between 62% and 86% of the eliminated horses. The robustness of these results was confirmed by high areas under the receiving operating characteristic curves (0.68–0.84). Overall, a horse has a 70% chance of being eliminated at the next gate if its cardiac recovery time is longer than 11 min at vet gate 1 or 2, or longer than 13 min at vet gates 3 or 4. Heart rate recovery and average speed variables measured at the previous vet gate(s) enabled us to predict elimination at the following vet gate. These variables should be checked at each veterinary examination, in order to detect unfit horses as early as possible. Our predictive method may help to improve equine welfare and ethical considerations in endurance events.

## Introduction

Endurance riding is an international, long-distance equestrian sport that has been regulated by the *Fédération Equestre Internationale* (FEI) since 1982. The first endurance world championships were held in 1998 in the United Arab Emirates (UAE). Since then, the number of entries in endurance events worldwide has increased more than four-fold [1]. Endurance events (ranging from 80 to 160 km in total distance) are split into successive phases of approximately 30–40km. At the end of each phase, horses are stopped for a veterinary inspection at the "vet gate". The heart rate (HR) is the primary criterion evaluated at the vet gate. For a horse to be considered fit enough to continue the event, its HR must be below 65 bpm within 20 minutes of arrival (although the exact thresholds vary according to the level of the event) [2]. During the recovery period, the support crews use various cooling techniques to shorten the HR recovery time and thus enable the horses to pass the veterinary inspection more quickly. It should be noted that the time interval between arrival at the vet gate and the start of the veterinary inspection is counted as part of the overall riding time. Consequently, rapid cardiac recovery is a key criterion for success in endurance events. Any horse deemed unfit to continue (due to lameness or excessive fatigue, for example) is immediately withdrawn from the event.

Elimination rates appear to have increased over recent years [3]. This a source of concern for the sport’s ethics and image. Lameness, dehydration and metabolic disorders are the main causes of elimination in 160 km events [4]. Elimination rates in endurance events vary from one geographical area to another [5]. For example, eliminations for metabolic disorders occur more frequently in hot and humid countries [6]. However, lameness remains the most common cause of elimination in all countries [5,7,8]. Several studies [3,8] have demonstrated that a combination of hard tracks, sudden changes in track surface and high speed might be associated with high elimination rates for lameness. A recent study of 4326 entries in major endurance events found that the mean elimination rate was 54%—primarily due to lameness (69.2%) and then metabolic reasons (23.5%) [8]. After a 160 km endurance event, an elevated hematocrit and low sodium, chloride and potassium concentrations were observed in both qualified and eliminated horses [9]. On the cardiac level, left ventricular systolic function appears to decrease in horses participating in endurance events [10]. Eliminated and qualified horses have been found to differ in terms of gene expression (particularly in leukocytes), and the clinical phenotype of eliminated horses is associated with an inflammatory/catabolic gene expression profile [11]. Given that a significant proportion (12%) of eliminated horses require emergency medical treatment, the rapid detection of tired horses is a challenge for veterinarians and a key issue in the improvement of equine welfare [3,12].

The HR is a good indicator of stress and health status in horses [13,14,15]. In thoroughbred horses, the HR measured 4 minutes after treadmill exercise was significantly correlated with Timeform ratings [16]. However, the relationship between performance horses and HR variables has not previously been characterized in endurance. Exercise training reduces recovery time by improving parasympathetic function after exercise and increasing aerobic capacity [17,18]. HR recovery after completion of each phase of the event is a key parameter for success in endurance events because the cardiac recovery time (CRT) is taken into account when calculating the average speed (AS) of each phase of the event [2].

Hence, we hypothesized that (i) qualified and eliminated horses differ in terms of their speed profiles and cardiac recovery variables, and (ii) this difference can be used to detect unfit horses and prevent health incidents during endurance events. The objective of the present study was to compute logistic regression models using the HR, CRT and AS recorded at the previous vet gate (n-1) and thus predict elimination at subsequent phases (vet gate n and following) of the event.

## Methods

### Data

**Fig 1:**
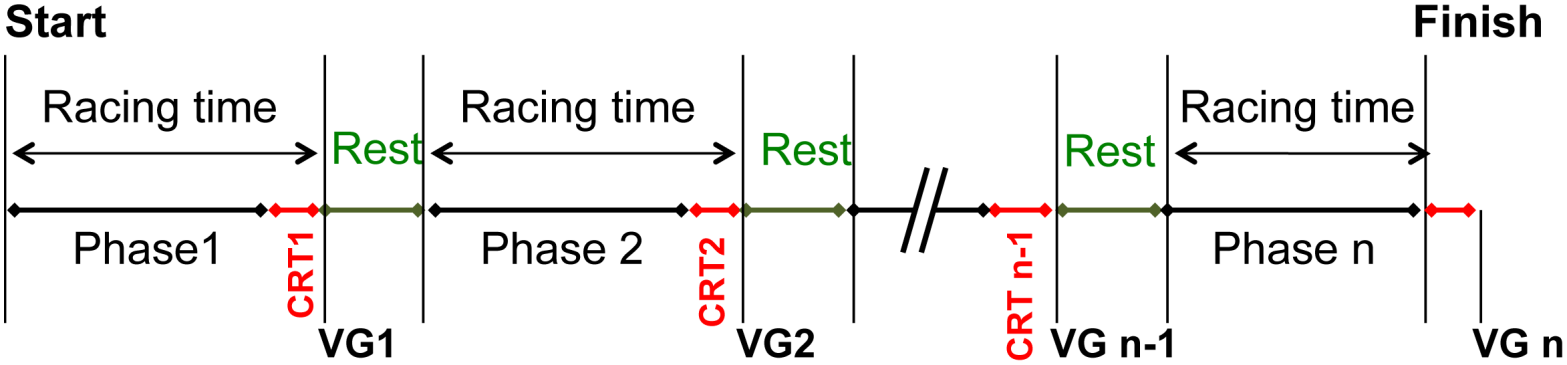
Schematic diagram of an endurance event. The competition is divided into phases of 30–40 km that are followed by 40- to 50-minute rest periods. Horses are checked by veterinarians at the vet gates (VGs) between each phase. The HR must be below 65 bpm when the horse is examined at the vet gate. The cardiac recovery time (CRT) is defined as time interval between the end of the phase and presentation at the vet gate; it counts towards the total running time.

Endurance events (ranging from 80 km to 160 km in total distance) are split into four, five or six successive phases of approximately 30–40 km. At the end of each phase, each horse undergoes a veterinary inspection (referred to as a vet gate; Fig 1).

The GenEndurance project (which encompasses the present study) was approved by the local Animal Care and Use Committee (ComEth Anses/ENVA/UPEC, Maisons-Alfort, France; approval number: 12/07/11-1). However, the present study did not involve any experiments on animals. All the horses having provided data for the present study had competed in official events organized according to the FEI rules [2]). All study data were extracted from the electronic timing records (ATRM Systems, Tarbes, France) of endurance events having taken place between 2007 and 2011 [19]). In total, the database included 4070 different horses and 7032 starts in high-level national and international endurance events over distances of 80 to 160 km (rated as 1-, 2- or 3-star events) (S1 Table). A total of 1622 horses participated in at least two events; the proportion of multi-starters did not differ significantly as a function of distance, country or the reason for elimination (S2 Table). The events took place in four countries: France (FRA, accounting for 78.7% of the event), the United Arab Emirates (UAE: 4.7%), and Spain+Portugal (SPA+POR, pooled data: 6.6%). Missing data for some horses (age, phase and reason for elimination) were retrieved from the FEI’s web database [1]. Two categorical variables were used to characterize the events in which HR and speed data were collected:

- the type of event, according to the distance ridden in one day: 1-star events (80 to 119 km: 27.2%), 2-star events (120 to 139 km: 54.9%) and 3-star events (140 to 160 km: 17.9%).
- the country in which the event was organized: FRA, SPA+POR and UAE. Climate data (temperature and humidity) for these zones were extracted *a posteriori* from a meteorological database and are given in S3 Table [20]. For the logistic regressions, data from SPA and POR were pooled because of the low number of events and the similar weather conditions in the two countries.

At each vet gate, the HR is measured by a veterinarian using a stethoscope. The CRT was calculated as the difference between the arrival time (at the end of the phase) and the time of veterinary inspection (referred to as the "time in" by the FEI endurance rules [2]). When two HR values were available for the same inspection, only the first value was considered for further analysis. The following variables were analyzed for all horses:

- the AS for each phase.
- the CRT and HR at each vet gate.
- if appropriate, the number of the vet gate at which the horse was eliminated and the reason for elimination. Horses can be elimination at any vet gate if the horse is considered to be unfit.
- event outcome: at the end of the event, the horses were divided in two groups: qualified (Q) horses had finished the event and passed all veterinary examinations, whereas eliminated (EL) horses had been eliminated by the judges for one of the four following reasons as defined in the following FEI endurance rules [2]:
  - **Lameness (LA),** for horses consistently observed to be lame by a panel of veterinarians. Lameness is “an irregularity of gait which must be consistently observable at trot, or an equivalent gait; and is observable through evaluation by trotting the horse on a loose lead in hand straight out and back, without prior flexion or deep palpation; which must be observed to cause pain, or threaten the immediate ability of the horse to safely perform athletically".
  - **Metabolic reasons (ME),** for horses that failed to achieve the required heart rate threshold (below 65 bpm, for example) required by the event’s distance and number of stars) or did not meet the criteria for over biological parameters (body temperature, blood pressure, hydration, electrolyte balance, intestinal activity, HR, etc.). Metabolic status is assessed by “the examination and recording of those parameters that indicate the horse’s fitness to continue including (but not exclusive to) mucous membranes, capillary refill time, hydration, intestinal activity, behavior and cardiac recovery index”.
  - **Retirement (RET),** for horses voluntarily withdrawn from event by their rider despite having passed all required veterinary inspections up to that point.
  - **Elimination for other reasons (OR),** for horses eliminated from the event for reasons other than those listed above.

Lastly, in order to check the validity of the predictive logistic regressions, we computed the probability of elimination for an independent dataset of 80 horses having competed in 1, 2 and 3-star endurance events organized in 2014. The 80 horses were randomly selected so that the dataset contained 10 qualified and 10 eliminated horses for each phase (i.e. phases 2, 3, 4 and 5).

### Statistical analysis

We used predictive logistic regression analysis to estimate probability of elimination from vet gates 2 to 5 and at the finish. The input variables were AS, CRT and HR measured at the previous vet gate (n-1). Given the absence of pre-ride data (CRT and HR at rest), horses eliminated at vet gate 1 (i.e. after the first phase of the event) were not included in the study. For each vet gate, an equal number of Q and EL horses were selected at random from the total set of available horses. Logistic regression consisted in computing the probability of be classified as qualified (Q) or eliminated (EL), using three explanatory variables (AS, CRT and HR) and two categorical variables (distance and country) as input variables. Briefly, the odds ratio was log-transformed (using the Newton-Raphson algorithm) in order to iteratively optimize the best model of the categorical and quantitative explanatory variables (using NCSS-2007 software [21,22]). In contrast to other classification methods (such as discriminate analysis), logistic regression does not require any assumptions to be made as to whether the explanatory variables are distributed normally or not. In order to quantify the regression’s predictive power for each phase, we measured the area under the receiving operating characteristic (ROC) curve (AUROC).

## Results

### Elimination of horses from endurance events

**Fig 2:**
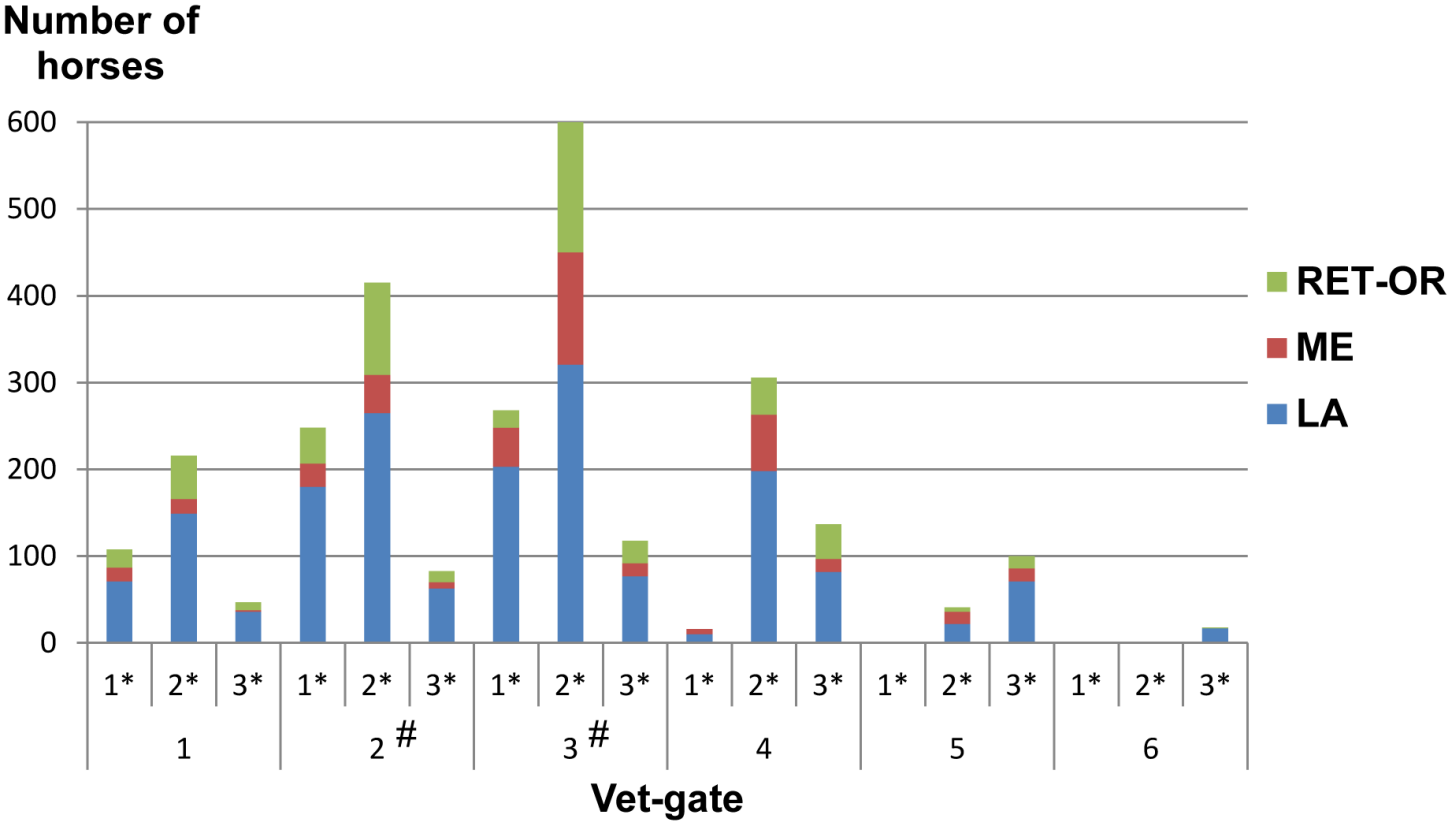
Distribution of the eliminated horses as a function of the cause of elimination, the vet gate and the event distance. 1*: 80–119 km, 2*: 120–139 km, 3*: 140–160 km, LA: lameness, ME: metabolic reasons, RET: retirement, OR: other reasons. # indicates a significantly greater elimination rate for all distance categories.

Of the 7032 starting horses, 38.94% (2738) were eliminated during an event; 64.46% of these eliminations were due to LA, with 15.23% due to ME, 14.35% due to RET and 5.96% due to OR (Table 1). Most eliminations (63.8%) occurred at vet gates 2 or 3, where the probability of elimination was higher in all events (regardless of the event distance) (Fig 2). The three speeds and cardiac recovery variables were strongly influenced by the outcome of the event (EL or Q) and the event distance (Table 2).

**Table 1:** Distribution of horses according to the outcome of the event and the reason for elimination.

	Event outcome		Reason for elimination			
Horses	Qualified	Eliminated	Lameness*	Metabolic disorders	Retired	Other reasons
Number	4294	2738	**1765**	417	393	163
Percentage	61.06%	38.94%	**64.46%**	15.23%	14.35%	5.96%

*Significant difference at p<0.05; **bold characters** = highest values.

**Table 2:** The AS, CRT and HR as a function of the outcome of the event, for different distance categories.

		Event outcome			
Performance	Distance category	Qualified (Q)	LA	ME	RET
Average	1-star event (#)	16.80 (2.31)	16.94 (2.44) ^ab^	17.24 (3.23) ^a^	16.32 (2.67) ^b^
speed (km/h)	2-star event	17.50 (3.01)	17.82 (3.21)	17.71 (3.35)	17.72 (3.75)
	3-star event	15.70 (1.83)	15.73 (1.52)	15.47 (1.63)	15.38 (1.79)
Cardiac	1-star event (#)	5.94 (2.89)	6.95 (3.36) ^b^	12.01 (7.00) ^a^	7.56 (3.92) ^b^
recovery time	2-star event (#)	4.77 (2.15)	5.01 (2.48) ^c^	7.99 (3.96) ^a^	6.66 (3.49) ^b^
(min)	3-star event (#)	4.25 (1.74)	4.61 (3.80) ^b^	6.82 (3.36) ^a^	5.77 (2.97) ^a^
Heart rate	1-star event (#)	55.77 (4.34)	55.21 (4.80) ^c^	61.93 (6.50) ^a^	57.02 (5.72) ^b^
(bpm)	2-star event (#)	58.03 (3.27)	57.90 (4.31) ^b^	61.41 (5.08) ^a^	57.97 (4.40) ^b^
	3-star event (#)	59.90 (2.11)	95.82 (3.6) ^b^	62.74 (3.69) ^a^	60.13 (3.24) ^b^

Data are presented as the mean (standard deviation) LA: lameness; ME: metabolic reasons; RET: retired; 1-star event: 80–115 km; 2-star event: 119–130 km, 3-star event: 140–160 km.

For each variable and event level marked by (#) an analysis of variance tested a significant effect between the reasons of elimination at p<0.05. The superscript letters ^a^, ^b^ and ^c^ indicate significant differences (p<0.05) between mean values for the various reasons of elimination.

### Predictive logistic regressions

**Fig 3:**
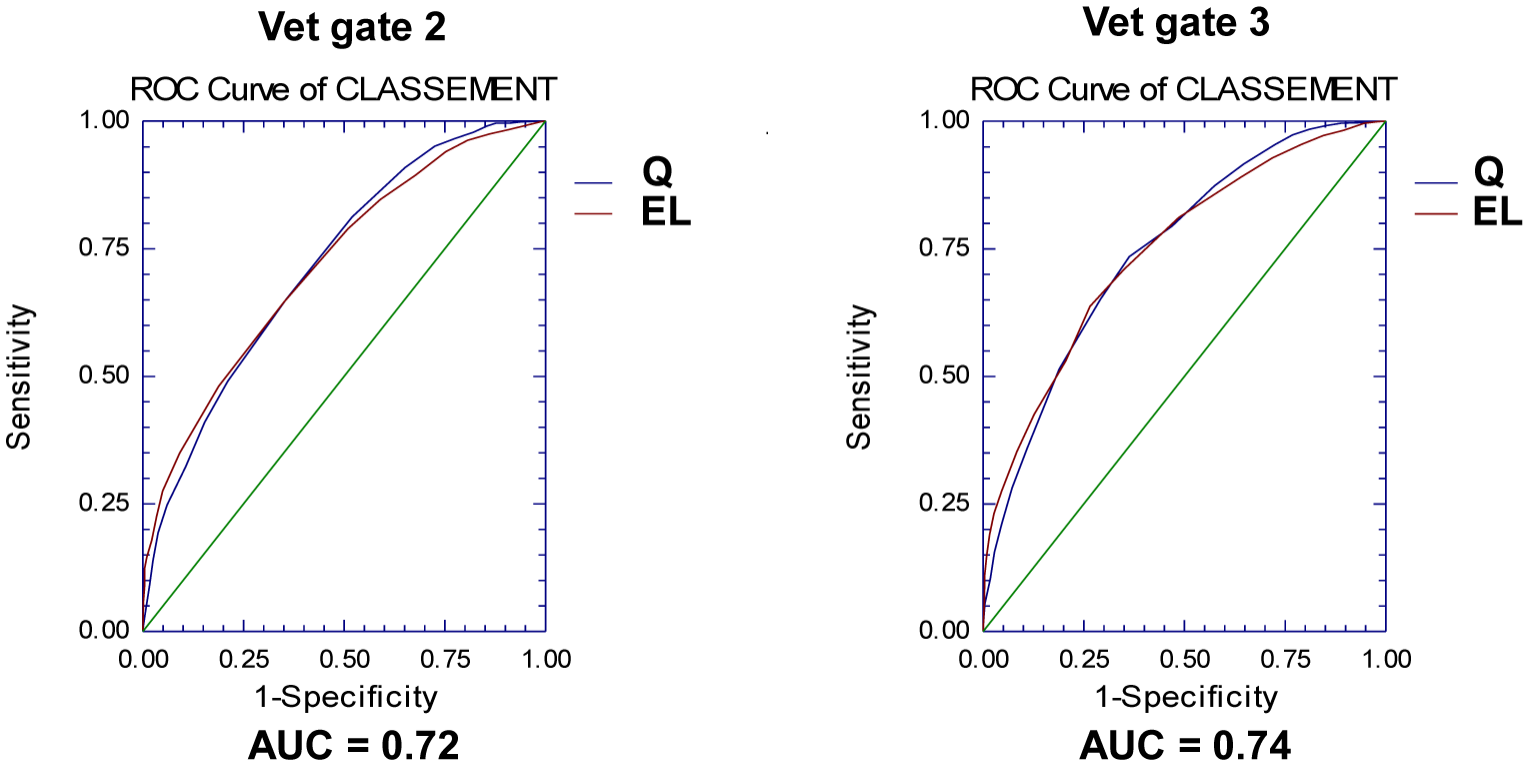
ROC curves of the logistic regressions for vet gates 2 and 3. The AUROC values (0.72 and 0.74, respectively) were acceptable and were associated with correct classification rates of 64% and 68%, respectively (AUROC = area under the ROC curve, Q = qualified, EL = eliminated).

The optimum logistic regression was computed for each vet gate (gates 2 to 5). The best indicators of validity are the percentage of well-classified observations and the AUROC, which characterize the logistic regression model’s ability to predict a horse’s classification as EL or Q (Table 3 and S4 Table). The percentage of correctly classified horses at each vet gate ranged from 61.8% to 86.6% (Table 3). The AUROC values ranged from 0.68 to 0.84 (relative to a maximum possible value of 1) and thus indicated good predictive power for classification of the horses at vet gates 2 to 5 (Table 3 and Fig 3). The predictive power was highest for vet gate 5 (with a correct classification rate of 86.6% and an AUROC of 0.84). In contrast to linear regression, the R^2^ value derived from logistic regression is not a good indicator of validity because it estimates the percentage of variation in the binary dependent variable (Q or EL) accounted for by the explanatory variables AS, CRT and HR at the previous vet gate (n-1). In the present study, R^2^ values were between 0.12 and 0.37; however these values did not accurately reflect the logistic regressions’ predictive power.

**Table 3:** Results of the logistic regressions used to estimate the probability of elimination at each vet gate (based on variables recorded at the previous vet gate).

Vet gate	Vet gate 2	Vet gate 3	Vet gate 4	Vet gate 5
Number of observations	1450	1961	874	253
Explanatory variables *	AS _1_, CRT _1_, HR _1_	AS _2_, CRT _2_, HR _2_	AS _3_, CRT _3_, HR _3_	AS _4_, CRT _4_, HR _4_
Categorical variables *	distance and age	distance and age	distance	distance
Percentage of horses correctly classified	64.80%	68.70%	61.80%	86.60%
AUROC	0.72	0.74	0.68	0.84
Estimated R^2^	0.12	0.15	0.10	0.37
Sensitivity (EL)	0.64	0.73	0.71	0.70
Specificity (EL)	0.64	0.63	0.54	0.99

*explanatory and categorical variables included in the logistic regression using the Newton-Raphson method.

AS_n-1_: average speed measured before vet gate n; CRT_n-1_: cardiac recovery time before vet gate n; HR_n-1_: heart rate measured before vet gate n; EL: eliminated; AUROC: area under the receiver operating characteristic curve.

Using the same dataset, we also computed logistic regressions to predict the reason for elimination (i.e. LA, ME, RET and OR) at each vet gate (S4 Table). The correct classification rate at each vet gate ranged from 51.18% to 73.5%; these values were lower than for Q vs. EL predictions.

### Validation of the predictive logistic regressions with an independent dataset

The ROC curves enabled us to determine the false positive rate and false negative rate with regard to the probability of elimination. When considering the horse’s health, it is better to have false positives than false negatives. A probability of 50% would have generated too many false negatives. Hence, we chose a probability of elimination of 70% as a threshold for classifying the predictions based on an independent dataset from 2014. Using this threshold, 75% of the 80 horses were classified correctly (Table 4). The error rate of 25% corresponded to a false negative rate of 8.7% and a false positive rate of 16.3%.

**Table 4:** Validation of the predictive logistic regressions by applying an independent dataset (including 80 horses having competed in endurance events in 2014). By applying a 70% probability of elimination in the regression calculations, the correct prediction rate was 75%. There were relatively few false negative predictions (8.70%), whereas the false positive rate was higher (16.30%).

	REAL STATUS		PREDICTED STATUS		PREDICTIONERROR		
Qualified	48	60%	35	43.70%	13	16.30%	False positive
Eliminated	32	40%	25	31.20%	7	8.70%	False negative
Total	80	100%	60	75%	20	25%	False prediction

### Detection of unfit horses via the definition of critical thresholds for HR recovery variables

**Fig 4:**
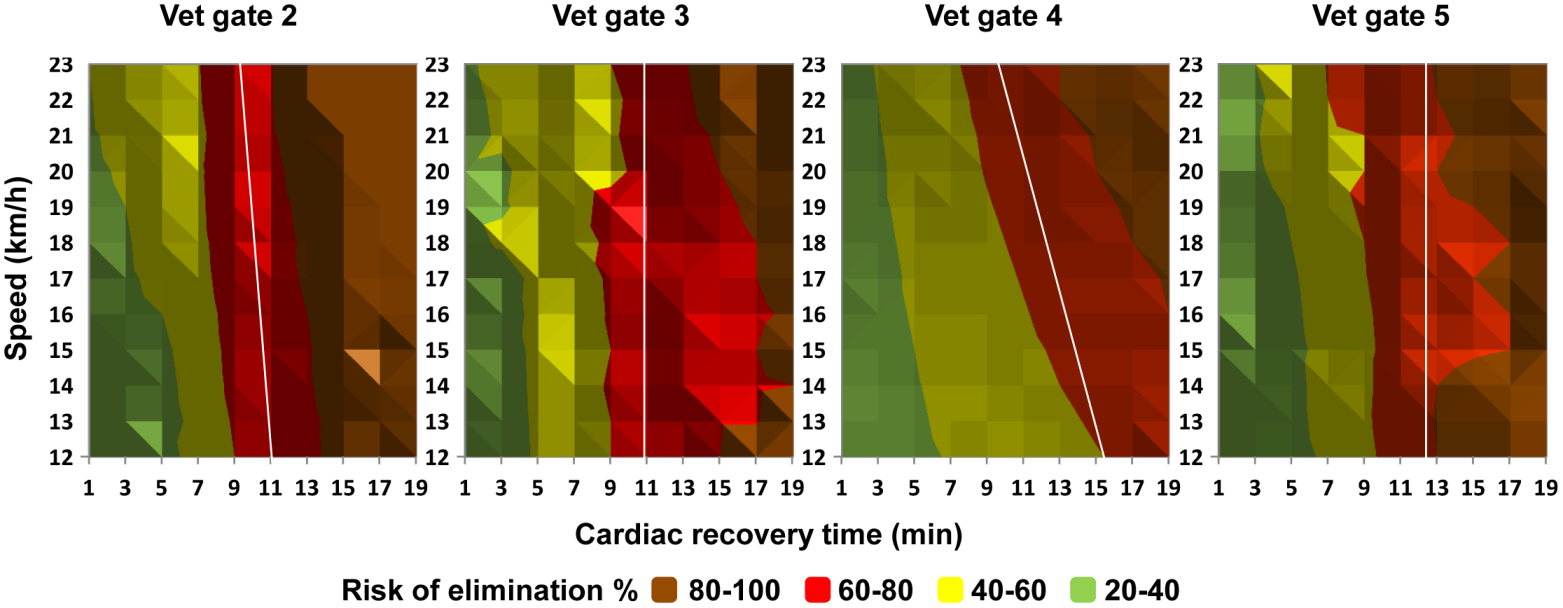
Three-dimensional scatter plots showing the probability of elimination at vet gates 2, 3, 4 and 5, according to the corresponding logistic regressions with a fixed HR of 64 and the AS and CRT measured at the previous vet gate (n-1). Red corresponds to a probability of elimination of 60 to 80%, whereas brown (the darkest areas) corresponds to a probability of 80 to 100%. The white line corresponds to a probability of elimination of 70% (the threshold chosen to compute the probability of elimination in an independent data set used for validation).

Lastly, we determined the threshold values of AS and CRT that corresponded to a 70% probability of elimination at a vet gate *n* due to an HR of 65 or more. Using the logistic regression equations with a HR value set to 64, we computed all the combinations of CRT and AS that gave a probability of elimination of between 20% and 100%. The results are presented using a colour scale in order to show the probability of elimination when the CRT and AS were above the computed thresholds at the previous vet gate (n-1) (Fig 4). Briefly, a horse that needs more than 11 min to recover (i.e. to achieve an HR below 65 bpm) at vet gates 1 or 2 has a 70% probability of being eliminated at vet gates 2 or 3 (Fig 4), respectively. At vet gates 3 or 4, a horse that needs more than 13 min to recover has a 70% probability of being eliminated at vet gates 4 or 5, respectively (Fig 4). Although the probability of elimination rises with increasing AS for all phases, the phenomenon is more pronounced in phases 2 and 3 (as observed when considering the relative distribution of eliminations during the events; Fig 2 and Table 2).

## Discussion

By analyzing a large dataset from equine endurance events (from 80 to 160 km in distance) organized in four different countries, we were able to compute logistic regressions and estimate the probability of elimination at each vet gate. On average, between 64.8 to 86.6% of all eliminations at vet gates 2 to 5 could be predicted from data collected at the previous vet gate. Furthermore, the predictive models’ robustness was confirmed with a new, independent dataset; 75% of the eliminations were correctly predicted. These results confirm the relationship between CRT, HR and AS measured at phase n-1 and elimination at phase n. Changes in CRT, HR and AS during the event are clearly associated with an increased probability of elimination at the next vet gate. The higher the speed, the higher the probability of elimination. This result is in line with previous studies [3,8]. The AS in phases 2 and 3 appears to be strongly associated with the probability of elimination at vetgates 3 and 4. Most metabolic changes occur during the first half of an event [4,23]; when combined with high speed, the effect of these changes increases the probability of elimination later in the event. Elevated values of CRT and HR were also associated with a greater probability of elimination [24].

The data collected in the present study corresponded to a quarter of the FEI-registered endurance events organized between 2007 and 2011. These data were more representative of the major events, which have a high enough budget to finance electronic time-keeping. Hence, our results might have been biased by the high-level events. Most of the events in the database took place in three European countries (SPA-PORT and FRA), with few events in the UAE. Other countries with major endurance events (the USA, New Zealand, Australia, South America countries, the UK, South Africa, Italy, etc.) were not included in the database. Strictly, the results presented here can be applied in FRA, ESP, PORT and UAE—the countries on which our regression models were based—and thus should be evaluated with caution in other countries (particularly those with a different climate and/or different course profiles. Twenty-three percent of the horses were represented twice or more in the database because they started in more than one event. However, participation in an event can be considered as being partially independent of the other events, since an event’s outcome depends on (among other things) the horse’s fitness and the prevailing environmental conditions (other competitors, the course profile, weather conditions, riding style and tactics, etc.) [5,8].

Although several studies [3,8] have highlighted the risks associated with excessively high speed in endurance events, the present study may be the first to have demonstrated the relationship between AS, CRT and the probability of elimination. Our study data showed that 39% of the horses were eliminated. The main reasons for elimination were lameness and then metabolic reasons; this was true in all four countries studied. The incidence of elimination measured in the present study falls within the range of values reported in the literature (from 18.9% [24] to 54% [8]). In the validation sample of horses having raced in 2014, the incidence of elimination (again 39%) agrees with epidemiological data for the general population of endurance horses [8, 24]. Differences in the reported incidence of elimination may be due to differences in the numbers of starts, weather conditions and/or the event distance. In studies of endurance events, lameness has always been the most frequent reason for elimination (accounting for between 50 and 70% of eliminations) [8,24]. High elimination rates in endurance events have been linked to several factors: the environmental conditions at the race site, a high number of starters in the event, long event distances, the track conditions, the training method and the riding style [5,8]. Breeds of horses with a high body mass index (such as the Appaloosa and the Quarter Horse) are more likely to be eliminated than purebred Arabians [24]. In summary, our sample was representative of the epidemiological data recorded in endurance events in Europe and the UAE. The data enabled us to compute logistic regression models for predicting the probability of elimination from events in these countries. Furthermore, our predictive logistic regressions took into account the main effects of the event level (distance) and the country in which the event took place (which includes weather conditions and other specific factors).

Eliminations for LA or MET are intended to prevent tired horses from continuing the event and then developing conditions associated with exhaustion and illness [4,25]. Despite the application of stringent rules and thorough veterinary examinations, it has been reported that 12% of the horses starting an endurance events require emergency treatment after wards [12]. Consequently, it is essential to identify horses likely to be eliminated as early as possible, in order to improve animal welfare, decrease the incidence of subsequent pathologies and avoid accidents during and after endurance events. The present study was designed to investigate the possible relationship between the probability of elimination and the measured performance-related variables (AS, HR and CRT). Our results showed that eliminated horses had higher HR values and a longer CRT at vet gates than qualified horses. This tachycardia might be a warning sign of peripheral and central fatigue. Tachycardia can result from a decrease in HR variability and low levels of parasympathetic activity, with persistence of the sympathetic component [26]. An increase in HR during events and a decrease in gastrointestinal sounds have already been associated with an increased probability of elimination for MET in endurance horses [24]. The HR is considered to be a good indicator of the degree of fatigue during endurance exercise; HR monitoring is therefore an important means of assessing fitness levels [18,27]. In humans, endurance training can increase parasympathetic nervous system activity and accelerate HR recovery [28,29]. In healthy people, HR recovery is an independent prognostic marker of cardiovascular disease and all-cause mortality [30]. Good training and efficient event management might limit fluid and electrolyte imbalances by improving the horses’ cardiac and aerobic capacities during the event [6,17,18,25,23].

Multiple regression, discriminant analysis and logistic regression can potentially all be used to classify horses as Q or EL as a function of the input variables available in event records. When discriminant analysis and logistic regression were compared, the latter method appeared to be the best for a two-group context (as in the present study) [31]. Furthermore, logistic regression does not require any specific assumptions concerning the distribution of the measured variables; this is not the case in factorial discriminant analysis, which assumes a normal data distribution. The results of our logistic regressions showed that it is possible to predict the elimination of a horse on the basis of the HR, CRT and AS measured at the previous vet gate (n-1). The number of observations used for each logistic regression was acceptable when seeking to compute accurate models of elimination in different types of high-level endurance events. The AUROC enabled us to quantify the false positive and false negative prediction rates, and is the best criterion for validating a model’s predictive power [32]. Surprisingly, the R^2^ obtained from a logistic regression analysis is not an appropriate measure of the quality of the binary model response because it is only an approximate value. This was confirmed in the present study because the low but acceptable R^2^ values (0.12–0.37) were associated with rather high AUROC values (0.68–0.84), which confirmed the model’s ability to distinguish between two groups. In the present study, we chose to favor a higher false positive rate over a high false negative rate, so that an unfit and potentially ill horse could be identified as early as possible. Hence, we chose a probability of elimination of 70% for the validation test, this corresponded to a false positive rate of 16.3% and a false negative rate of only 8.7%. It was not possible to include more explanatory variables (such as weather conditions or the horse’s age) in the models because the small sample size and unequal number of Q and EL horses in each subgroup would have prevented high-quality prediction. Including a putative effect of double starts in the statistical models was not possible because it would have generated too many the degrees of freedom for robust calculations. We also computed also logistic regressions to predict the reason for elimination at each vet gate, however the correct prediction rate was too low for most of the vet gates (except for the last vet gate 5, where few horses were eliminated).

Lastly, the present study made it possible to calculate CRT threshold values that could be usefully applied to examinations at the vet gate: horses requiring more than 11 min to achieve an HR below 65 at vet gates 1 and 2 or more than 13 min at vet gates 3 and 4 have a 70% chance of being eliminated at the next vet gates (2, 3, 4 and 5). This information has great practical value and suggests that it would be possible to detect tired horses earlier in the event than veterinarians currently do. It has been demonstrated that the current vet gate examination enables veterinarians to detect physiologically compromised horses [2,4]. However, the horses’ health could be safeguarded by detecting and eliminated unfit animals as early as possible in the event, i.e. before the appearance of patent signs of exhaustion. Hence, the CRT, the recovery check and other clinical measurement appear to be particularly useful markers of fitness [7,24]. Application of the CRT threshold values calculated in the present study (11 and 13 minutes) might help to reduce the morbidity seen at endurance event and therefore improve equine welfare in this extreme sport. However, eliminations are likely to be the end result of a multifactorial process; only the veterinarians will be able to integrate all types of information (CRT, HR, AS and other clinical variables). Given the statistical nature of the prediction and the presence of false positive predictions, the present predictive models should not be used directly as a FEI rule for eliminating a horse but could serve as a tool for improving the efficiency of the vet gates. For example, the probability of elimination could be given to the rider and the trainer as a warning. They could therefore decide whether to retire, slow down (in order to decrease the risk of elimination at the next vet gate) or maintain the same racing strategy (despite the high risk of elimination at the next vet gate).

## Conclusion

Our results show that it is possible to compute predictive logistic regressions for each vet gate (2 to 5) and correctly detect between 61.8 and 86.6% of horses subsequently judged to be unfit, by using AS, CRT and HR measured at the previous vet gate). The CRT appears to be a particularly relevant predictive variable for detecting unfit horses during the event. We suggest that the CRT should be checked at each vet gate, in order to identify unfit horses earlier and thus improve their welfare. In practice, CRT threshold values of 11 min (at vet gates 1 and 2) and 13 min (at vet gates 3 and 4) are associated with a 70% probability of elimination at the next vet gate. We encourage the veterinarians involved in endurance events and the FEI to apply this new knowledge, in order to improve the welfare of endurance horses and prevent health problems during or after endurance events.

## Supporting Information

S1 Table Total raw data including 7032 performance records used in the present study. (XLSX)

S2 Table Distribution of horses according to the number of starts, number of eliminations, reason for elimination, distance category and country. (PDF)

S3 Table Mean (SD) shade temperature (°C) and relative humidity (%) in each country, for the month and the season during which the endurance event was organized. (PDF)

S4 Table Results of the logistic regression, with an estimation of the probability of elimination as a function of the event outcome and the reason for elimination at each vet gate (based on variables recorded at the previous vet gate). (PDF)
