# Supplementary material for: Speed and Cardiac Recovery Variables Predict the Probability of Elimination in Equine Endurance Events
Source: PLoS One. 2015 Aug 31;10(8):e0137013. doi: 10.1371/journal.pone.0137013 (PMC4556447; doi:10.1371/journal.pone.0137013)
Supplement: S2 Table — (PDF) [file pone.0137013.s002.pdf]

Table S2: Distribution of horses according to the number of starts, number of eliminations, reason for elimination, distance categories and country where the race has been organized.

Number of starts<br>N = **7032**	Q	EL	Reason for elimination				Distance category of eliminations			Country of eliminations		
			LA	ME	RET	OR	1*	2**	3***	UAE	FRA	SPA-POR
1	543	269	715	196	134	60	945	1257	246	621	1610	217
2	1031	666	442	89	98	37	500	962	235	273	1311	113
3	1343	1105	280	75	68	29	252	763	296	108	1124	79
4	859	452	175	29	46	19	123	471	218	30	750	32
5	158	88	62	14	18	9	54	188	108	2	340	8
6	247	103	56	5	20	7	26	132	88	0	236	10
7	18	6	27	9	9	2	8	67	51	0	122	4
8	79	47	6	0	0	0	2	13	9	0	24	0
9	16	2	2	0	0	0	1	5	12	0	17	1
Total	4294	2738	1765	417	393	163	1911	3858	1263	1034	5534	464

Q: qualified; EL: Eliminated; OR: Other Reasons; LA: Lameness; ME: Metabolic; RET: Retirement. Distance categories - 1* = 80-119 km; 2* = 120-139 km; 3* = 140-160. SPA-POR: Spain-Portugal; UAE: United Arab Emirates. FRA: France.
