# Supplementary material for: Speed and Cardiac Recovery Variables Predict the Probability of Elimination in Equine Endurance Events
Source: PLoS One. 2015 Aug 31;10(8):e0137013. doi: 10.1371/journal.pone.0137013 (PMC4556447; doi:10.1371/journal.pone.0137013)
Supplement: S3 Table — (PDF) [file pone.0137013.s003.pdf]

Table S3: Mean (SD) shade temperature (°C) and relative humidity (%) in each country, for the month and the season during which the endurance event was organized.

Country *	Season*	Temperature (°C)	Humidity (%)	Number of starts
FRA [a]	Autumn	14 ± 4 [b']	62 ± 13	1024
	Spring	17 ± 4 [a']	61 ± 11	2063
	Summer	19 ± 3 [b']	64 ± 10	2447
ESP [a]	Spring	16 ± 4 [a']	57 ± 9	303
	Summer	23 ± 5 [b']	60 ± 8.5	19
	Winter	14 ± 6 [b']	70 ± 6	33
POR [a]	Autumn	25 ± 5 [b']	50 ± 12	64
	Spring	16 ± 6 [a']	65 ± 10	45
UAE [b]	Autumn	28 ± 3	48 ± 6	444
	winter	21 ± 2	46 ± 5	590

Means ± standard-deviation (sd), calculated over a period of one month from endurance competition. ESP-POR: Spain-Portugal; UAE: United Arab Emirates. FRA: France. Significant effect of country and the season of competitions; the means followed by different superscript letters are significantly different at $P<0.05$: a, b, c for mean test within country and a', b' c' for mean test within Season. There is no significant effect of the season on Humidity.
