# Supplementary material for: Speed and Cardiac Recovery Variables Predict the Probability of Elimination in Equine Endurance Events
Source: PLoS One. 2015 Aug 31;10(8):e0137013. doi: 10.1371/journal.pone.0137013 (PMC4556447; doi:10.1371/journal.pone.0137013)
Supplement: S4 Table — (PDF) [file pone.0137013.s004.pdf]

Table S4: Results of the logistic regression, with an estimation of the probability of elimination as a function of the event outcome and the reason for elimination at each vet gate (based on variables recorded at the previous vet gate).

Veterinary examinations	Vet gate 2	Vet gate 3	Vet gate 4	Vet gate 5
Number of observations	1151	1961	874	113
Explanatory variables*	AS_1<br>CRT_1<br>HR_1	AS_2<br>CRT_2<br>HR_2	AS_3<br>CRT_3<br>HR_3	AS_4<br>CRT_4<br>HR_4
Categorical variable *	Outcome of the competition (Q, LA, ME, RET, OR)	Outcome of the competition (Q, LA, ME, RET, OR)	Outcome of the competition (Q, LA, ME, RET, OR)	Outcome of the competition (Q, LA, ME, RET, OR)
Percentage of horses correctly classified	63.90%	54.10%	51.10%	73.50%
AUC of ROC, Means (SD)	0.58 (0.02)	0.60 (0.04)	0.62 (0.07)	0.78 (0.02)
Estimated R^2	0.02	0.04	0.06	0.25

*explanatory and categorical variables included in the logistic regression by the Newton-Raphson method. AS_{n-1}: average speed measured before vet gate n; CRT_{n-1}: cardiac recovery time before Vet gate n; HR_{n-1}: heart rate measured before vet gate n; Q: qualified; LA: lameness; ME: metabolic disoroders; RET: .retired; OR: other reasons; AUC: Area under curve; ROC: Receiver operating characteristic.
